# Role of gut microbiota in postoperative complications and prognosis of gastrointestinal surgery: A narrative review

**DOI:** 10.1097/MD.0000000000029826

**Published:** 2022-07-22

**Authors:** Yong Shi, Huxiao Cui, Fangjie Wang, Yanxia Zhang, Qingbin Xu, Dan Liu, Kunhui Wang, Sen Hou

**Affiliations:** a Department of General Surgery, Xuchang Central Hospital, Xuchang City, Henan Province, China.

**Keywords:** complications, gut microbiota, gastrointestinal surgery, Probiotics

## Abstract

Gastrointestinal surgery is often challenging because of unexpected postoperative complications such as pouchitis, malabsorption, anastomotic leak, diarrhea, inflammatory responses, and life-threatening infections. Moreover, the gut microbiota has been shown to be associated with the complications described above. Major intestinal reconstruction, such as Roux-en-Y gastric bypass (RYGB) and ileal pouch-anal anastomosis surgery, could result in altered gut microbiota, which might lead to some of the benefits of these procedures but could also contribute to the development of postsurgical complications. Moreover, postsurgical reestablishment of the gut microbiota population is still poorly understood. Here, we review evidence outlining the role of gut microbiota in complications of gastrointestinal surgery, especially malabsorption, anastomotic leak, pouchitis, and infections. In addition, this review will evaluate the risks and benefits of live biotherapeutics in the complications of gastrointestinal surgery.

## 1. Introduction

More than ten years after the launch of the Human Microbiome Project, related studies on the role of microorganisms in infectious and complex human diseases have become the research focus of researchers worldwide.^[1]^ However, the application of microbial communities (bacterial, fungal, and viral communities) in the fields of medicine and human health remains challenging.^[2]^ The following 3 processes are required to translate microbiome science into clinical practice: First, we should find evidence of the contribution of the microbiome to disease progression in clinical practice. Second, direct mechanistic studies need to be carried out using biochemical, immunological, or microbiological approaches to explore the mechanism of specific microbial strains in disease progression. Third, clinical interventions (e.g., fecal microbiome transplantation, medication, diet, probiotics, or prebiotics) or large clinical trials need to be conducted to further clarify the role of specific microbial strains in disease.^[1,2]^ Only by following this “clinical observation-directed mechanistic studies-clinical intervention” principle can we translate microbiome science into clinical practice and achieve the ultimate goal of precision medicine.^[3]^

In the past few decades, with the progress in gastrointestinal surgery technology and clinical care, various postoperative complications have decreased significantly.^[2]^ Postoperative infection can lead to increased mortality, delayed rehabilitation, and increased medical cost.^[4]^ Consequently, surgeons and infection control officers are actively looking for a variety of novel approaches to reduce infectious complications.^[5]^ More and more research showed that gut microbiota was closely related with the infectious complications of gastrointestinal surgery, such as pouchitis, surgical site infection, intraabdominal abscess, and postoperative inflammatory responses.^[6–8]^ Traditional wisdom suggests that decontamination can eliminate postoperative infection.^[9]^ However, it is impossible to completely eradicate intestinal bacteria during the preparation for gastrointestinal surgery, and it may also lead to adverse complications. *Clostridium difficile* is an anaerobic bacterium that usually lives in the human intestine. If antibiotics are taken excessively, the growth rate of Clostridium difficile will accelerate, which will affect other bacteria in the intestine and cause pseudomembranous enteritis and diarrhea.^[3,10,11]^ Under normal conditions, the human intestinal microbiota is critical for the host’s resistance to both endogenous and exogenous pathogens.^[2]^ In addition, intestinal microorganisms can activate immune cells and trigger an immune response.^[3]^ Therefore, maintaining the balance of the gut microbiota is of great significance for the rapid recovery of patients undergoing gastrointestinal surgery.

For severe ulcerative colitis (UC) and familial adenomatous polyposis (FAP), total colectomy combined with ileal pouch-anal anastomosis (IPAA) is often performed for severe ulcerative colitis (UC) and familial adenomatous polyposis. Roux-en-Y gastric bypass (RYGB) surgery is often performed to treat obesity. Major intestinal reconstructions, such as RYGB and IPAA surgery, can alter the gut microbiota.^[2–4]^ On one hand, altered gut microbiota might lead to some of the benefits of these procedures. However, they can also contribute to the development of postsurgical complications.^[2]^ Live biotherapeutics (including FMT and probiotics) could generate some benefits, such as restoring the diversity of damaged intestinal microorganisms and contributing to clinical resolution in recurrent *Clostridium difficile-associated* diarrhea.^[3]^ However, some risks associated with live biotherapeutics have recently been identified. For instance, probiotics can cause septicemia in some compromised patients.^[4]^ In this review, we will comprehensively summarize the relationship between gut microbiota and complications of gastrointestinal surgery, including colorectal cancer surgery, gastrectomy, IPAA, and RYGB surgery, which may be of great significance in preventing complications of gastrointestinal surgery.

## 2. Gut microbiota is involved in pathogenesis of gastrointestinal cancer

**Figure 1.**
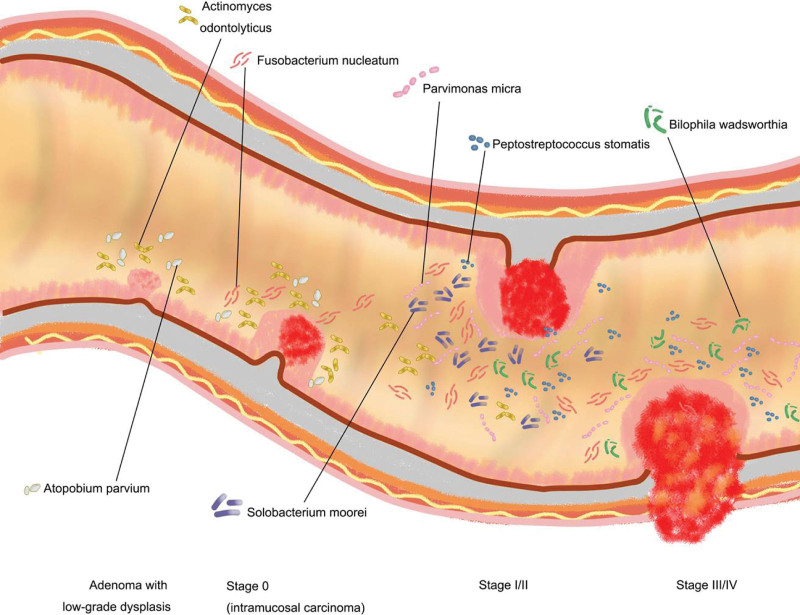
Graphical representation of major change of microbial species during multistep CRC progression.

Intestinal microorganisms are more than just local colonization bystanders. They perform various biological functions to maintain human health.^[2]^ Symbiotic bacteria actively maintain epithelial barrier function. Second, these symbiotic bacteria contribute to the development of the host immune system (e.g., innate immunity and T-helper-cell function) and play a key role in the prevention of infectious complications. Changes in the gut ecosystem are associated with human diseases including gastrointestinal cancer. For example, many studies have shown evidence of a series of changes in major fecal microorganisms during multistep colorectal cancer (CRC) progression.^[5]^ These distinct stage-specific phenotypes of fecal microorganisms were further identified using a whole-genome shotgun metagenomic approach. Specifically, some sulfide-producing bacteria, such as *Bilophila wadsworthia, Desulfovibrio vietnamensis*, and *D. longreachensis*, were significantly elevated in stage III/IV (SIII/IV) CRC than in stage I/II (SI/II) CRC. In addition, some newly identified CRC-related species (e.g., *Streptococcus anginosus*, *Porphyromonas uenonis, Colinsella aerofaciens*, and *Selenomonas sputigena*) were also found to be elevated in SIII/IV CRCs than in SI/II. Interestingly, 2 butyrate producers (e.g., *Eubacterium eligens* and *Lachnospira multipara*) were significantly depleted at all CRC stages,^[5]^ indicating that microbial changes in multiple species are possibly related to the multistep process of colorectal carcinogenesis (Fig. 1).

## 3. Preoperative preparation and the gut microbiota

Ohigashi et al proposed the theory that a poorly diversified microbiome is more likely to develop disease than a well-diversified microbiome.^[6]^ Some preoperative preparations, such as purgative cleansing and oral and intravenous antibiotics, may cause major shifts in the bacterial composition of a poorly diversified microbiome, providing an opportunity for pathogenic bacteria to repopulate the lumen.^[6]^ Therefore, significant changes in the intestinal environment might be associated with postoperative complications.

## 4. Surgical stress and gut microbiota

There is much evidence that surgery itself can cause a significant change in the composition of the gut microbiota.^[2]^ For instance, colectomy in rats results in a significant increase in mucosal-associated lumen bacteria, including *Escherichia* and *Enterococcus*. Moreover, altered microbial communities at the ileostomy site were observed in patients who underwent small-bowel transplantation. Changes in the gut microbiota include an increase in facultative anaerobes (e.g., *Enterobacteriaceae* and *Lactobacillus*) and the depletion of obligate anaerobes (e.g., *Clostridia* and *Bacteroides*). A possible reason for this is that exposure of the intestinal lumen to the ambient atmosphere changed its original anaerobic environment. In addition, ischemia-reperfusion injury caused by ligation of intestinal blood vessels during surgery is also an important reason for the change in gut microbiota. An animal experiment further demonstrated that ischemia-reperfusion injury caused by ligation of the superior mesenteric artery might lead to an increase in the abundance of *E. coli* and a decrease in *Lactobacillus* in the rat ileum and colon.^[7]^

## 5. Host–microorganism communication

Research has shown that bacteria have a strong ability to sense local environmental signals and determine their population density through quorum sensing, which greatly improves their adaptability to the surrounding environment.^[2]^ The hospital-acquired and commensal microbiota can sense the host state through inflammatory signals (e.g., IL-6, IL-8, IFN-γ, various cytokines) and hormones that are secreted into the lumen during surgery. In general, this adaptive progression of pathogens is terminated by modern surgical treatment, including the proper use of antibiotics, adequate nutrition, and timely liquid therapy.^[2]^ However, strong surgical stressors caused by long surgery duration and large injury are sensed by pathogenic bacteria (e.g., *E. faecalis* or *P. aeruginosa*), which may lead to pathogen overgrowth to adapt to harsh environmental conditions. In this case, the postoperative complications may have increased significantly. Therefore, it is important to take active measures to promote postsurgical microbiota recovery and refaunation.^[2]^ Recently, the concept of enhanced recovery after surgery (ERAS) has become widely used. ERAS emphasizes postoperative rehabilitation treatment, including early mobilization, enteral nutrition, and discontinuation of opioid analgesia. However, it is still unclear whether it promotes postsurgical microbiota recovery and refaunation, which requires further exploration in the future.

## 6. Changes of gut microbiota after gastrointestinal surgery

Next, we discuss the changes in the gut microbiota after gastrointestinal surgery, including colorectal and gastrointestinal surgeries.

### 6.1. Colorectal surgery

Changes in the gut microbiota of patients with CRC before and after surgical treatment have been confirmed in many studies.^[8]^ Recently, genomic data of 28 patients with CRC before and nearly 1 year after surgery showed that the relative abundance of oral anaerobes, such as *Parvimonas micra, Peptoanaerobacter stomatis, Peptostreptococcus anaerobius, Dorea longicatena*, and *Porphyromonas uenonis*, were dramatically reduced after tumor removal.^[9]^ Notably, these strains have been identified as marker species for CRC. Likewise, Ohigashi et al also demonstrated that the number of obligate anaerobes, such as *bifidobacteria*, was significantly reduced after surgery.^[6]^ In contrast, the number of pathogenic bacteria, such as *Enterobacteriaceae, Enterococcus, Staphylococcus*, and *Pseudomonas*, increases significantly after surgery.^[6]^

### 6.2. Gastrectomy and alimentary reconstruction

**Figure 2.**
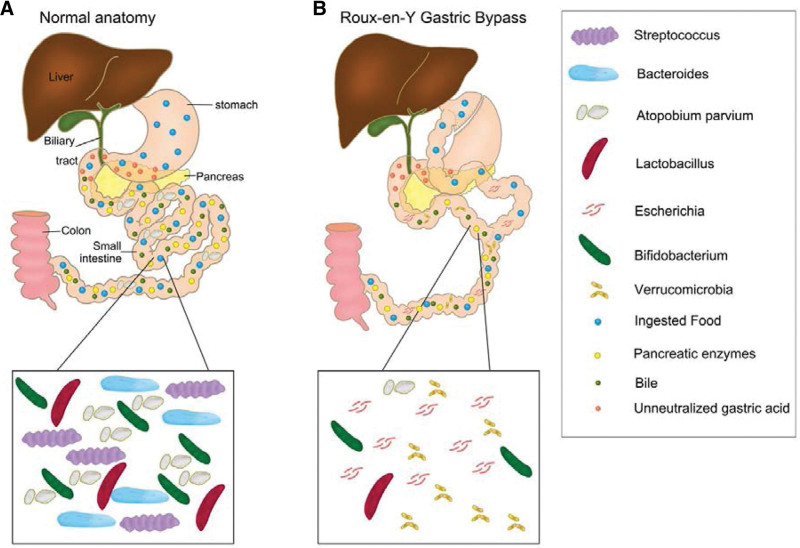
Anatomical reconstructions lead to the changes in physiological function and composition of gut microbiota. (A) Normal anatomy. (B) Roux-en-Y gastric bypass.

Currently, it is generally believed that digestion and absorption functions depend on the gut microbiota. Each part of the gut has a unique symbiotic ecosystem that facilitates the decomposition, processing, and absorption of various nutrients. Therefore, surgical reconstruction of the gastrointestinal tract fundamentally affects the downstream microecological balance and then affects digestion, absorption, and immune function. Many of the surgical reconstructions used today are based on technology developed decades ago.^[2]^ In the early 20th century, surgeons treated recurrent ulcers using distal gastrectomy. As time went by Billroth I, Billroth II, and Roux-en-Y reconstruction were performed to restore intestinal continuity.^[2]^ These operations keep biliopancreatic secretions away from most of the alimentary canal and contact only the distal jejunum, ileum, or colon (Fig. 2). RYGB has been used to treat morbid obesity owing to its weight loss effect. Over the past decade, the effect of RYGB on the gut microbiota has been well studied. In obesity and associated diabetes, *Firmicutes* abundance was higher, but *Bacteroides* abundance was lower, while the ratio of *Firmicutes/Bacteroides* was significantly decreased in patients after RYGB.^[10]^ Likewise, a recent study demonstrated rapid and lasting changes in gut microbiota in the mouse gastrointestinal tract after RYGB operation, especially an increased abundance of *Escherichia* and *Verrucomicrobia* (Fig. 2).^[10]^ Surprisingly, when the RYGB-associated microbiota was transplanted into germ-free mice without gastrointestinal tract reconstruction (sham control), it also led to significant weight loss and reduced body fat compared to those that received microbiota transfer from sham surgery mice.^[11]^ This further confirmed that the changes in microbiota caused by RYGB surgery play an important role in weight loss and metabolic changes.

## 7. Gut microbiota and postoperative long-term oncological outcomes

Many studies have confirmed that higher levels of *Fusobacterium nucleatum* (*F. nucleatum*) in colorectal tumor samples are correlated with worse outcomes, in particular, decreased overall survival, disease-free survival, recurrence-free survival, and cancer-specific survival rates.^[5–10]^ Mechanistically, *F. nucleatum* promotes chemoresistance in colorectal cancer by activating the autophagy pathway.^[5]^ Moreover, *F. nucleatum* may accelerate the growth of tumor cells and weaken T cell-mediated immune responses against colorectal tumors.^[6]^ In contrast, Flemer et al found that tissue-associated microbial co-abundance groups (CAGs), namely Prevotella-CAGs and Pathogen-CAGs (including *F. nucleatum*), are associated with improved survival in patients with CRC.^[11]^ This may be due to the fact that many of the studied individuals have been followed up for less than 2 years. Moreover, some confounding factors affecting oncological outcomes were not adjusted in this study. In addition to *F. nucleatum*, 1 study showed that the abundance of *Bifidobacterium* was correlated with the extent of signet ring cells. However, no statistically significant correlation between the concentration of *Bifidobacterium* and overall mortality was observed in this study.^[12]^

## 8. Gut microbiota influences postoperative complications of gastrointestinal surgery

Complications caused by complex gastrointestinal reconstructive surgery can affect patient recovery. These complications usually include pouchitis, malabsorption, inflammation, and anastomotic leaks. Numerous studies have shown that there may be a close relationship between the gut microbiota and the development of postoperative complications.

### 8.1. Pouchitis

Pouchitis is one of the most common complications of radical correction in patients with ulcerative colitis (UC). Approximately 10% to 35% of UC patients require surgery because of their resistance to standard therapy.^[12]^ Approximately 40% of these patients develop pouchitis within 1 year of surgery. Several risk factors for pouchitis have been reported, including primary sclerosing cholangitis, nonsteroidal antiinflammatory drug (NSAID) use, preoperative thrombocytopenia, and positive antineutrophil cytoplasmic antibody. Accumulating evidence suggests that intestinal dysbacteriosis may occur in pouchitis and plays a key role in disease progression.^[13]^ Moreover, it has been reported that antibiotics and probiotics have a good effect of ameliorating the symptoms of pouchitis, further suggesting that bacterial translocation may play an important role in the generation and development of pouchitis. For example, 1 study showed that the pouch microbial environment seems to be distinctly different in the settings of pouchitis, healthy pouches, ulcerative colitis (UC), and familial adenomatous polyposis (FAP).^[14]^ Moreover, Komanduri et al found that inflamed pouch mucosa had greater bacterial species diversity than healthy pouch mucosa.^[13]^ Furthermore, using 16S rDNA sequencing, Zella et al revealed that the pouchitis group had fewer *Bacteroidetes* and *Proteobacteria* and more *Firmicutes, Clostridia*, and *Verrucomicrobia than* the healthy FAP group.^[14]^ Several bacterial species have been reported to play important roles in the progression of pouchitis, but their potential in disease screening remains to be explored. Most recently, Machiels et al reported that some specific members of the primary microbial community could predict pouchitis in UC patients undergoing colectomy within the 1st year after ileal pouch-anal anastomosis (IPAA).^[15]^ More specifically, a risk score model for pouchitis based on the presence of *Ruminococcus gnavus, Bacteroides vulgatus*, and *Clostridium perfringens* and the absence of *Blautia* and *Roseburia* in fecal samples of patients with UC before surgery was established. Higher scores were associated with a higher likelihood of developing pouchitis after IPAA.^[15]^ Therefore, it is of great significance to analyze the microbial components in feces before colectomy to screen patients who may develop pouchitis, which may lead to new predictive and therapeutic strategies.

### 8.2. Malabsorption

Complex reconstructive procedures such as RYGB, sleeve gastrectomy, or pancreatoduodenectomy may cause a series of complications, such as reflux esophagitis, diarrhea, dumping syndrome (fast stomach emptying), anemia, osteoporosis, fat malabsorption, and vitamin B12 deficiency.^[2]^ Notably, these processes have a greater impact on energy metabolism through the gut microbiota. It was found that these reconstructive procedures could increase the number of bacteria involved in glucose uptake in the small intestine, such as *Akkermansia spp.*, and decrease the number of bacteria involved in bile acid metabolism, such as *Bifidobacterium spp*.^[16]^ Likewise, Patrone et al revealed that the abundance of *Lactobacillus* was negatively associated with patients’ blood glucose levels after correcting for confounding factors, such as caloric intake.^[17]^ In addition, the abundance of *Roseburia* species is directly associated with host metabolism because of its ability to ferment a variety of carbohydrates. Moreover, the increase in *Escherichia coli (E.coli*) is related to the higher energy acquisition efficiency in postRYGB starvation-like conditions. Most recently, Furet et al reported that high levels of *Gammaproteobacteria* were closely related to malabsorption after RYGB.^[18]^ These findings reinforce the tight links between gut microbiota and carbohydrate metabolism after RYGB. The risk of malnutrition (e.g., trace metal and vitamin deficiencies) is high in patients after RYGB surgery. Fortunately, most patients who experience this malabsorption gradually recover through nutritional counseling and vitamin supplementation. In the future, a deeper exploration of the mechanisms linking gut microbiota and malabsorption in patients who have undergone digestive tract reconstruction could facilitate precise therapies for this complication.

### 8.3. Inflammation pathology

Compelling evidence suggests that *Faecalibacterium prausnitzii* (*F. prausnitzii*) is closely related to inflammatory markers and low-grade inflammation after bariatric surgery.^[18]^ *F. praussnitzii* is considered a conserved and dominant species of fecal microbiota in healthy people, preventing inflammation and infection in acute inflammatory bowel disease. Moreover, the level of *F. prausnitzii* is closely associated with decreased low-grade inflammation and higher insulin resistance in patients with obesity and type 2 diabetes mellitus. Many studies have shown that the abundance of *F. prausnitzii* is low in patients with obesity or type 2 diabetes prior to bariatric surgery. However, the level of *F. prausnitzii* increased again after RYGB, which could reduce the level of low-grade inflammation.^[19]^ Mechanistically, the metabolites of *F. praussnitzii* can prevent the production of inflammatory mediators and activation of nuclear factor-kB. Some studies have shown that oral administration of *F. praussnitzii* or *F. praussnitzii* culture supernatant can increase the level of IL-10 and reduce circulating inflammatory parameters (e.g., C-reactive protein, IL-6, IL-12, and orosomucoid).^[18]^ In the future, *F. prausnitzii* may serve as a valuable therapeutic target for improving inflammatory disorders and insulin resistance after gastrointestinal surgery.

### 8.4. Anastomotic leak

**Figure 3.**
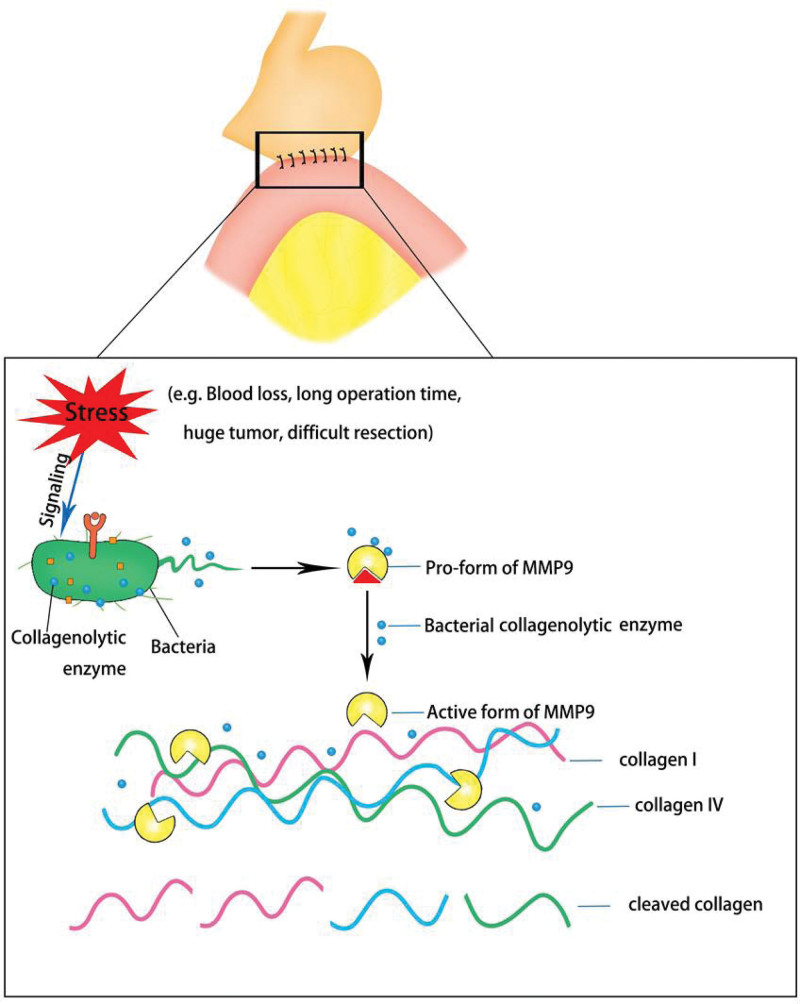
Hypothesis on the mechanism of anastomotic leak. The surgical stressors caused by long surgery time, blood loss, difficult resection or large injury will be sensed by the pathogenic bacteria, which may lead to the elevated collagenase production.

As one of the most devastating complications of gastrointestinal surgery, anastomotic leaks have plagued surgeons for decades, and there are no better preventive measures. Although surgical techniques and postoperative care have improved over the years, anastomotic leakage often occurs with serious consequences such as morbidity, diverting stomas, and fatal infections. More than 60 years ago, animal experiments performed on dogs fully proved that intestinal microorganisms play an important role in the occurrence of anastomotic leakage. In 1 study, transverse colostomy was performed, and the supplying blood vessels at the anastomotic site were ligated to cause gross ischemia. Subsequently, a feeding tube was placed near the anastomotic site, and saline or antibiotics were administered to the colonic lumen via a tube placed near the anastomosis.^[20]^ The results showed that saline-treated dogs developed severe intestinal leakage and peritonitis, whereas in the antibiotic-treated group, the blood supply returned to normal and the anastomoses tended to heal. Subsequent experiments on rats also proved that microorganisms are the cause of anastomotic leakage.^[21]^ Moreover, some prospective, randomized, double-blind clinical trials have proven the effectiveness of antibiotic application in the prevention of esophagojejunal anastomotic leakage after total gastrectomy.^[22]^ Subsequent studies confirmed that high collagenase-producing intestinal microbes, such as fecal *Pseudomonas aeruginosa* and *Escherichia coli*, were the main causes of anastomotic leakage in both rats and humans. Olivas et al validated the hypothesis that the phenotypic transformation of bacteria settled at the anastomotic site from a general phenotype to a destroying phenotype that expresses high tissue collagenase could lead to anastomotic leakage.^[23]^ Genotype analysis revealed that a single nucleotide polymorphism mutation in the mexT gene was present in the *Pseudomonas aeruginosa* strain retrieved from leaking anastomotic tissues. In this study, the rats were exposed to preoperative fractionated radiation, as in cancer surgery. As the most common pathogen in the gut after exposure to radiation, *Pseudomonas aeruginosa* was inoculated in the intestine of rats that underwent low colorectal resection and 1-stage anastomosis. Only rats exposed to radiation and intestinal *Pseudomonas aeruginosa* colonization developed clinical anastomotic leakage.^[23]^ In conclusion, this model shows that some pathogenic microorganisms, such as *Pseudomonas aeruginosa*, can change their phenotypes in vivo to form a more pathogenic phenotype and cause intestinal leakage. In addition, another pathogenic microorganism, *Enterococcus faecalis (E. faecalis*), has been identified as the causal agent of anastomotic leak. Olivas et al validated the hypothesis that *E. faecalis* strains were involved in the course of anastomotic leak through their increased collagen-degrading activity by activating tissue matrix metalloproteinase 9 (MMP9) in host anastomotic tissues (Fig. 3).^[24]^ Moreover, anastomotic leakage could be prevented by inhibiting the activation of MMP9 or by eliminating *the Enterococcus faecalis* strain by local antibiotics directly applied to the intestinal tissue of rats.^[24]^ Also needed is a greater understanding of anastomotic leak from the perspective of microorganisms to integrate microbial science into surgical thought and practice.

## 9. The effects of prebiotics or probiotics on postoperative complications of gastrointestinal surgery

### 9.1. The effects of prebiotics or probiotics on colorectal surgery

Recent studies have established the functional importance of prebiotics or probiotic in postoperative complications of gastrointestinal surgery (including colorectal resection and RYGB surgery). To investigate the conclusiveness of the currently available evidence of prebiotics or probiotics on postoperative complications of colorectal resection surgery, we searched all the relevant literature to date. A total of 10 randomized controlled trials was ultimately included in this review.^[25–34]^ The features extracted from these studies were shown in Table 1. These clinical trials were published over the past 12 years with a sample size ranging from 31 to 379. For postoperative patients with CRC, probiotics have proven to be extremely important in reducing postoperative complications, such as surgical site infection,^[25–28]^ diarrhea, ^[28,29]^ septicemia,^[30,31]^ anastomotic leakage,^[25]^ bacterial translocation,^[32]^ inflammatory responses,^[33]^ ileus,^[25]^ intraabdominal abscess,^[25]^ etc. Moreover, patients treated with probiotics had improved quality of life and fewer days of postoperative hospitalization compared to patients untreated with probiotics,^[34]^ we consider that it would be very necessary to introduce routine use of probiotics in patients after colon and rectum cancer surgery.

**Table 1:** The outcomes of different clinical trials assessing the probiotics efficacy on colorectal cancer treatment.

References	Study type	Country	Intervention	Outcomes
Bajramagic^[25]^	RCT	Bosnia and Herzegovina	Starting from the third postoperative day lasting for the next 30 days	Probiotic has a significant reduction in postoperative complications (anastomosis loosening, surgical site infection, ileus, intraabdominal abscess) in CRC surgery.
Aisu^[26]^	RCT	Japan	six tablets/day	Probiotic treatment can reduce surgical site infection and improve the intestinal microbial environment in patients undergoing CRC surgery.
Wei^[27]^	RCT	China	3 times a day, 3 days preoperatively	Perioperative probiotics treatment could reduce infectious complications
Liu^[28]^	RCT	China	6 days preoperatively and 10 days	The postoperative recovery of peristalsis, incidence of diarrhea, and infectious-related complications
Yang^[29]^	RCT	China	5 days before and 7 days after CRC resection operation	The days to first flatus and first defecation significantly improved in the probiotic-treated patients. The incidence of diarrhea was significantly lower in probiotics group
Liu^[30]^	RCT	China	6 days preoperatively and 10 days postoperatively	Perioperative probiotics treatment could reduce the serum zonulin level, the rate of postoperative septicemia and maintain the liver barrier in patients undergoing CRC surgery
Liu^[31]^	RCT	China	6 days preoperatively and 10 days postoperatively	Perioperative probiotics treatment could reduce serum zonulin concentrations and the rate of postoperative septicemia
Reddy^[32]^	RCT	UK	Probiotic preparation was Trevis capsules 3 times daily	Synbiotics reduces the prevalence of fecal Enterobacteriaceae and bacterial translocation
Consoli^[33]^	RCT	Brazil	at least 7 days before surgery	Probiotic treatment with *S. boulardii* downregulates both pro- and antiinflammatory cytokines in the intestinal colonic mucosa
Tan^[34]^	RCT	Malaysia	Twice daily for a consecutive 7 days prior to surgery	Perioperative probiotics treatment lead to faster recovery and shorter duration of hospital stay

### 9.2. The effects of prebiotics or probiotics on RYGB bypass surgery

Regarding the studies on the evaluation of probiotics or prebiotics supplementation in gastrectomy and RYGB bypass surgery,^[35–38]^ the results showed that supplementation of *C. butyricum* and *B. longum* could reduce gastrointestinal symptoms and improve the quality of life of patients receiving gastrectomy and Roux-en-Y gastric bypass reconstructive surgery (Table 2). Furthermore, daily intake of 2.4 bi *Lactobacillus sp.* provides better results in bacterial overgrowth, and effectiveness of vitamin B12 synthesis after RYGB.^[35]^ Moreover, it has already been observed that a significant increase of serum vitamin B12 levels via synthesis by gut microbiota among individuals following supplementation with probiotics. In addition, the increase of vitamin D synthesis by gut microbiota was also observed in a study by Karbaschian et al.^[36]^ These studies showed that probiotics supplementation was beneficial to the maintenance of vitamin homeostasis in patients after RYGB operation. However, 1 study reported that probiotics administration could not improve hepatic inflammatory and clinical outcomes 6 and 12-months after RYGB.^[37]^ Moreover, Fernandes et al found that 1 × 10^9^ daily supplement of *L. paracasei, L. rhamnosus, L acidophilus, B. lactis* and with 6 g fructooligosaccharide for 15 days did not show any superior results in both placebo and prebiotic groups.^[38]^ There are 2 possible explanations for the negative results of these 2 trials. On the one hand, RYGB surgery has a significant impact on anthropometry, liver, inflammation and metabolic parameters, including microbial components, so the benefits of probiotic therapy will not outweigh the impact of surgery itself.^[37]^ On the other hand, RYGB surgery will cause the acceleration of gastric emptying and intestinal transport, while reducing the acid production. These conditions may affect the passage and survival of probiotics post RYGB surgery. Currently, it is still unclear whether a prophylactic regimen using probiotic could prevent the occurrence of complications in gastrectomy and Roux-en-Y gastric bypass surgery. In the future, large scale and multicenter randomized controlled trials need to be carried out as early as possible.

**Table 2:** The outcomes of different clinical trials assessing the probiotics efficacy on Roux-en-Y gastric bypass treatment.

References	Study type	Country	Year	Number of participants	Age range	Intervention	Outcomes
Woodard^[35]^	RCT	USA	2009	44	Not reported	6-month postoperatively	Probiotic administration improves bacterial overgrowth, vitamin B12 availability, and weight loss after RYGB.
Karbaschian^[36]^	RCT	Iran	2018	46	18–60	Pre 4 weeks Post 12 weeks	Probiotic supplementation promotes inflammatory markers, body weight loss, and status of vitamin D in patients undergoing RYGB.
Sherf-Dagan^[37]^	RCT	Israel	2018	100	41.9 ± 9.8	3-months	Probiotics administration does not improve hepatic, inflammatory and clinical outcomes 6- and 12-months post- RYGB.
Fernandes^[38]^	RCT	Brazil	2016	26	18–65	30 days after partial gastrectomy for up to 15 days	Prebiotics and synbiotics were not able to promote significant changes in inflammatory markers.

### 9.3. Existing problems of the modulation of gut microbiota in patients after gastrointestinal surgery

**Figure 4.**
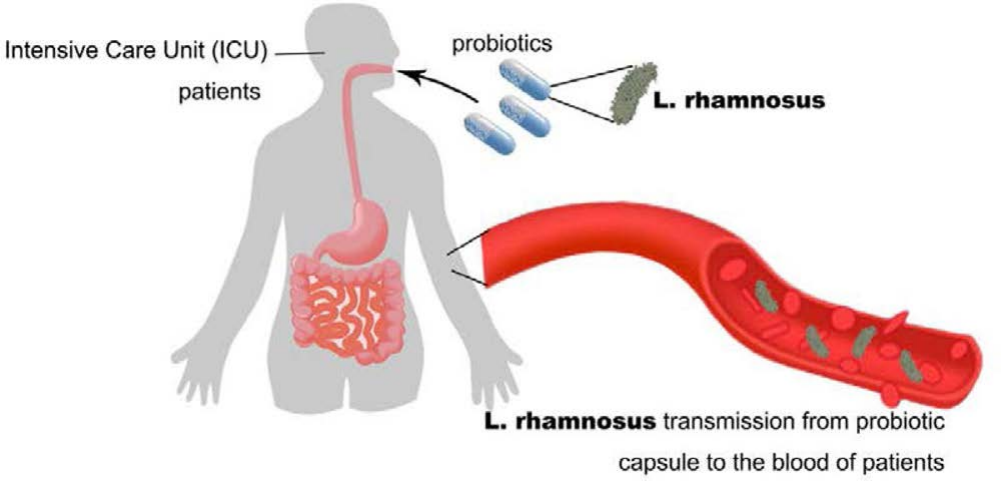
Bacterial transmission from probiotic capsule to blood in patients who are critically ill.

Postsurgical modulation of gut microbiota is crucial for improving the prognosis of patients undergoing gastrointestinal surgery.^[39–43]^ Although probiotics have been widely recognized for their beneficial health effects on the host and have a long record of safety for traditional generally recognized as safe strains,^[44–46]^ the potential risks associated with introducing living microorganisms into immunocompromised individuals using probiotics have recently been reported. Yelin et al reported that probiotic strains can directly cause *Lactobacillus* bacteremia in 6 patients in the intensive care unit at Boston Children’s Hospital.^[3]^ In this study, prebiotics containing *Lactobacillus* were used as a part of the treatment. Whole-genome sequencing clearly showed that the *lactobacilli* isolated from the blood of these 6 young patients were phylogenetically inseparable from the probiotics used.^[3]^ However, how these bacteria translocate from the gastrointestinal tract to the bloodstream remains unclear. Accordingly, the benefits and risks of probiotic treatment should be calculated in patients after gastrointestinal surgery, especially in the intensive care unit (Fig. 4).

## 10. Balancing the risks and rewards of live biotherapeutics

Clinically, both defined probiotics and undefined fecal microbiome transplantation are live biotherapeutics.^[47,48]^ The benefits and risks associated with probiotics in gastrointestinal surgeries are discussed in detail in the content presented above. Fecal microbiota transplantation (FMT) has been proven to be effective in improving or even curing diseases such as recurrent *Clostridium difficile* infection (CDI).^[49,50]^ In 2013, van Nood et al^[1]^ reported the results of a single-center trial on the treatment of CDI using duodenal infusion of donor feces. In this study, 43 patients were randomly divided into 3 groups: FMT, oral vancomycin, and vancomycin plus intestinal lavage groups. The results showed that the cure rate of CDI in the FMT group was significantly higher than those in the oral antibiotics and antibiotics plus intestinal cleaning groups. However, it is noteworthy that risks associated with FMT have recently been reported. For instance, DeFilipp et al found that FMT transferred extended-spectrum β-lactamase-producing Escherichia coli to 2 high-risk patients, both of whom received FMT capsules from the same stool donor.^[3]^ Given the possibility of FMT-related transmission of severe viral infections or multidrug-resistant bacteria, the clinical safety of FMT needs to be further studied. However, to date, the prevention of complications associated with gastrointestinal surgery using FMT has not been documented. Before using microbiome intervention for the management of postoperative complications of gastrointestinal surgery, we should better understand the role of gut microbiota in the occurrence, maintenance, and development of diseases.

## 11. Conclusions

Major intestinal reconstruction could result in altered gut microbiota, which might lead to some of the benefits of these procedures (e.g., improvement of inflammation disorders and insulin resistance) but could also contribute to the development of postsurgical complications (e.g., malabsorption and diarrhea). This suggests that the gut microbiota has a double-sided effect on the complications of gastrointestinal surgery. Although FMT has been proven to be effective in improving or even curing some diseases, such as recurrent *CDI*, the prevention of complications of gastrointestinal surgery by FMT has not been documented and needs further exploration in the future. ERAS emphasizes the need for postoperative rehabilitation. However, it is still unclear whether it promotes postsurgical microbiota recovery and refaunation, which requires further exploration in the future.

### Acknowledgment

We thank Professor Chaoxu Liu for revising this manuscript.

## References

[1] HarkinsCPKongHHSegreJA. Manipulating the human microbiome to manage disease. JAMA. 2019;17:E1–E2.10.1001/jama.2019.1960231876898

[2] Guyton K Alverdy JC. The gut microbiota and gastrointestinal surgery. Nat Rev Gastroenterol Hepatol. 2017;141:43–54. 10.1038/nrgastro.2016.139 27729657

[3] Hill C. Balancing the risks and rewards of live biotherapeutics. Nat Rev Gastroenterol Hepatol. 2019;17:133667–134. 10.1038/s41575-019-0254-3 31873192

[4] Yelin I Flett KB Merakou C. Genomic and epidemiological evidence of bacterial transmission from probiotic capsule to blood in ICU patients. Nat Med. 2019;25:1728–32. 31700189 10.1038/s41591-019-0626-9 PMC6980696

[5] Yachida S Mizutani S Shiroma H. Metagenomic and metabolomic analyses reveal distinct stage-specific phenotypes of the gut microbiota in colorectal cancer. Nat Med. 2019;25:968–76. 31171880 10.1038/s41591-019-0458-7

[6] Ohigashi S Sudo K Kobayashi D. Significant changes in the intestinal environment after surgery in patients with colorectal cancer. J Gastrointest Surg. 2013;17:1657–64. 23807702 10.1007/s11605-013-2270-x

[7] Wang F Li Q He Q. Temporal variations of the ileal microbiota in intestinal ischemia and reperfusion. Shock. 2013;39:96–103. 23247126 10.1097/SHK.0b013e318279265f

[8] Feng Q Liang S Jia H. Gut microbiome development along the colorectal adenoma-carcinoma sequence. Nat Commun. 2015;6:6528. 25758642 10.1038/ncomms7528

[9] Yachida S Mizutani S Shiroma H. Metagenomic and metabolomic analyses reveal distinct stage-specific phenotypes of the gut microbiota in colorectal cancer. Nat Med. 2019;25:968–76. 31171880 10.1038/s41591-019-0458-7

[10] Lutz TA Bueter M. The physiology underlying Roux-en-Y gastric bypass: a status report. Am J Physiol Regul Integr Comp Physiol. 2014;307:R1275–91. 25253084 10.1152/ajpregu.00185.2014 PMC4315446

[11] Liou AP Paziuk M Luevano JM. Conserved shifts in the gut microbiota due to gastric bypass reduce host weight and adiposity. Sci Transl Med. 2013;5:178ra41. 10.1126/scitranslmed.3005687 PMC3652229 23536013

[12] Landy J Al-Hassi HO McLaughlin SD. Etiology of pouchitis. Inflamm Bowel Dis. 2012;18:1146–55. 22021180 10.1002/ibd.21911

[13] Komanduri S Gillevet PM Sikaroodi M. Dysbiosis in pouchitis: evidence of unique microfloral patterns in pouch inflammation. Clin Gastroenterol Hepatol. 2007;5:352–60. 17368235 10.1016/j.cgh.2007.01.001

[14] Zella GC Hait EJ Glavan T. Distinct microbiome in pouchitis compared to healthy pouches in ulcerative colitis and familial adenomatous polyposis. Inflamm Bowel Dis. 2011;17:1092–100. 20845425 10.1002/ibd.21460

[15] Machiels K Sabino J Vandermosten L. Specific members of the predominant gut microbiota predict pouchitis following colectomy and IPAA in UC. Gut. 2017;66:79–88. 26423113 10.1136/gutjnl-2015-309398

[16] Miyachi T Nagao M Shibata C. Biliopancreatic limb plays an important role in metabolic improvement after duodenal-jejunal bypass in a rat model of diabetes. Surgery. 2016;159:1360–71. 26767308 10.1016/j.surg.2015.11.027

[17] Patrone V Vajana E Minuti A. Postoperative Changes in Fecal Bacterial Communities and Fermentation Products in Obese Patients Undergoing Bilio-Intestinal Bypass. Front Microbiol. 2016;7:200. 26941724 10.3389/fmicb.2016.00200 PMC4762995

[18] Furet JP Kong LC Tap J. Differential adaptation of human gut microbiota to bariatric surgery-induced weight loss: links with metabolic and low-grade inflammation markers. Diabetes. 2010;59:3049–57. 20876719 10.2337/db10-0253 PMC2992765

[19] Li M Wang B Zhang M. Symbiotic gut microbes modulate human metabolic phenotypes. Proc Natl Acad Sci USA. 2008;105:2117–22. 18252821 10.1073/pnas.0712038105 PMC2538887

[20] Fry DE. Colon preparation and surgical site infection. Am J Surg. 2011;202:225–32. 21429471 10.1016/j.amjsurg.2010.08.038

[21] Schardey HM. Bacteria: a major pathogenic factor for anastomotic insufficiency. Antimicrob Agents Chemother. 1994;38:2564–7. 7872748 10.1128/aac.38.11.2564 PMC188242

[22] Schardey HM Joosten U Finke U. The prevention of anastomotic leakage after total gastrectomy with local decontamination. a prospective, randomized, double-blind, placebo-controlled multicenter trial. Ann Surg. 1997;225:172–80. 9065294 10.1097/00000658-199702000-00005 PMC1190646

[23] Olivas AD Shogan BD Valuckaite V. Intestinal tissues induce an SNP mutation in Pseudomonas aeruginosa that enhances its virulence: possible role in anastomotic leak. PLoS One. 2012;7:e44326. 22952955 10.1371/journal.pone.0044326 PMC3432121

[24] Shogan BD Belogortseva N Luong PM. Collagen degradation and MMP9 activation by enterococcus faecalis contribute to intestinal anastomotic leak. Sci Transl Med. 2015;7:286ra 68. 10.1126/scitranslmed.3010658 PMC5027898 25947163

[25] Bajramagic S Hodzic E Mulabdic A. Usage of probiotics and its clinical significance at surgically treated patients suffering from colorectal carcinoma. Med Arch. 2019;73:316–20. 31819304 10.5455/medarh.2019.73.316-320 PMC6885229

[26] Aisu N Tanimura S Yamashita Y. Impact of perioperative probiotic treatment for surgical site infections in patients with colorectal cancer. Exp Ther Med. 2015;10:966–72. 26622423 10.3892/etm.2015.2640 PMC4533173

[27] Zhang JW Du P Gao J. Preoperative probiotics decrease postoperative infectious complications of colorectal cancer. Am J Med Sci. 2012;343:199–205. 22197980 10.1097/MAJ.0b013e31823aace6

[28] Liu Z Qin H Yang Z. Randomised clinical trial: the effects of perioperative probiotic treatment on barrier function and post-operative infectious complications in colorectal cancer surgery – a double-blind study. Aliment Pharmacol Ther. 2011;33:50–63. 21083585 10.1111/j.1365-2036.2010.04492.x

[29] Yang Y Xia Y Chen H. The effect of perioperative probiotics treatment for colorectal cancer: short-term outcomes of a randomized controlled trial. Oncotarget. 2016;7:8432–40. 26824990 10.18632/oncotarget.7045 PMC4885004

[30] Liu Z Li C Huang M. Positive regulatory effects of perioperative probiotic treatment on postoperative liver complications after colorectal liver metastases surgery: a double-center and double-blind randomized clinical trial. BMC Gastroenterol. 2015;15:34. 25881090 10.1186/s12876-015-0260-z PMC4374379

[31] Liu ZH Huang MJ Zhang XW. The effects of perioperative probiotic treatment on serum zonulin concentration and subsequent postoperative infectious complications after colorectal cancer surgery: a double-center and double-blind randomized clinical trial. Am J Clin Nutr. 2013;97:117–26. 23235200 10.3945/ajcn.112.040949

[32] Reddy BS Macfie J Gatt M. Randomized clinical trial of effect of synbiotics, neomycin and mechanical bowel preparation on intestinal barrier function in patients undergoing colectomy. Br J Surg. 2007;94:546–54. 17443852 10.1002/bjs.5705

[33] Consoli ML da Silva RS Nicoli JR. Randomized clinical trial: impact of oral administration of saccharomyces boulardii on gene expression of intestinal cytokines in patients undergoing colon resection. JPEN J Parenter Enteral Nutr. 2016;40:1114–21. 25917895 10.1177/0148607115584387

[34] Tan CK Said S Rajandram R. Pre-surgical administration of microbial cell preparation in colorectal cancer patients: a randomized controlled trial. World J Surg. 2016;40:1985–92. 27098538 10.1007/s00268-016-3499-9

[35] Woodard GA Encarnacion B Downey JR. Probiotics improve outcomes after Roux-en-Y gastric bypass surgery: a prospective randomized trial. J Gastrointest Surg. 2009;13:1198–204. 19381735 10.1007/s11605-009-0891-x

[36] Karbaschian Z Mokhtari Z Pazouki A. probiotic supplementation in morbid obese patients undergoing One Anastomosis Gastric Bypass-Mini Gastric Bypass (OAGB-MGB) Surgery: a randomized, double-blind, placebo-controlled, clinical trial. Obes Surg. 2018;28:2874–85. 29725975 10.1007/s11695-018-3280-2

[37] Sherf-Dagan S Zelber-Sagi S Zilberman-Schapira G. Probiotics administration following sleeve gastrectomy surgery: a randomized double-blind trial. Int J Obes (Lond). 2018;42:147–55. 28852205 10.1038/ijo.2017.210

[38] Fernandes R Beserra BT Mocellin MC. Effects of prebiotic and synbiotic supplementation on inflammatory markers and anthropometric indices after roux-en-y gastric bypass: a randomized, triple-blind, placebo-controlled pilot study. J Clin Gastroenterol. 2016;50:208–17. 25909598 10.1097/MCG.0000000000000328

[39] Guo Y Liu CQ Shan CX. Gut microbiota after Roux-en-Y gastric bypass and sleeve gastrectomy in a diabetic rat model: Increased diversity and associations of discriminant genera with metabolic changes. Diabetes Metab Res Rev. 2017;33:340–7. 10.1002/dmrr.2857 27572277

[40] Osto M Abegg K Bueter M. Roux-en-Y gastric bypass surgery in rats alters gut microbiota profile along the intestine. Physiol Behav. 2013;119:92–6. 23770330 10.1016/j.physbeh.2013.06.008

[41] Dawoodbhoy FM Patel BK Patel K. Gut microbiota dysbiosis as a target for improved post-surgical outcomes and improved patient care: a review of current literature. 2021;55:441–54. 10.1097/SHK.0000000000001654 32881759

[42] Lee JA Chico TJA Renshaw SA. The triune of intestinal microbiome, genetics and inflammatory status and its impact on the healing of lower gastrointestinal anastomoses. 2018;285:1212–25. 10.1111/febs.14346 PMC5947287 29193751

[43] Mo S Ru H Huang M. Oral-Intestinal microbiota in colorectal cancer: inflammation and immunosuppression. 2022;15:747–759. 10.2147/JIR.S344321 PMC8824753 35153499

[44] Suez J Zmora N Segal E. The pros, cons, and many unknowns of probiotics. Nat Med. 2019;25:716–29. 31061539 10.1038/s41591-019-0439-x

[45] Wilkins T Sequoia J. Probiotics for gastrointestinal conditions: a summary of the evidence. Am Fam Physician. 2017;96:170–8. 28762696

[46] Williams NT. Probiotics. Am J Health Syst Pharm. 2010;67:449–58. 20208051 10.2146/ajhp090168

[47] Tan Y Shen J Si T. Engineered live biotherapeutics: progress and challenges. Biotechnol J. 2020;15:e2000155. 32770635 10.1002/biot.202000155

[48] O’Toole PW Marchesi JR Hill C. Next-generation probiotics: the spectrum from probiotics to live biotherapeutics. Nat Microbiol. 2017;2:17057. 28440276 10.1038/nmicrobiol.2017.57

[49] Juul FE Garborg K Bretthauer M. Fecal microbiota transplantation for primary clostridium difficile infection. N Engl J Med. 2018;378:2535–6. 29860912 10.1056/NEJMc1803103

[50] Hvas CL Dahl Jørgensen SM Jørgensen SP. Fecal microbiota transplantation is superior to fidaxomicin for treatment of recurrent clostridium difficile infection. Gastroenterology. 2019;156:1324–1332.e3. 30610862 10.1053/j.gastro.2018.12.019

